# Eye-hand coordination during upper limb motor tasks in individuals with or without a neurodevelopmental disorder: a systematic review

**DOI:** 10.3389/fneur.2025.1569438

**Published:** 2025-06-25

**Authors:** Manel Abid, Isabelle Poitras, Martine Gagnon, Catherine Mercier

**Affiliations:** ^1^Center for Interdisciplinary Research in Rehabilitation and Social Integration (Cirris), CIUSSS De La Capitale-Nationale, Quebec, QC, Canada; ^2^School of Rehabilitation Sciences, Faculty of Medicine, Université Laval, Quebec, QC, Canada; ^3^Library, Université Laval, Quebec, QC, Canada

**Keywords:** developmental delay, motor deficits, visuomotor integration, oculomanual tracking, sensorimotor skills

## Abstract

**Background:**

Individuals living with neurodevelopmental disorders (NDD) often face challenges in performing daily manual activities that require precise visuomotor coordination. This systematic review aimed to characterize the differences between individuals with and without NDD in spatiotemporal eye-hand coordination when performing upper limb (UL) motor tasks.

**Methods:**

The following databases were systematically searched: CINAHL Plus with Full Texts (EBSCOhost), EMBASE.com, WEB OF SCIENCE core collection, All Ovid MEDLINE(R) and ERGONOMICS ABSTRACTS (EBSCOhost) in December 2022 and re-searched in April 2024. The studies selection was performed independently by two researchers according to the following inclusion criteria: (1) individuals diagnosed with NDD; (2) inclusion of aged-matched control (CTRL) group; and (3) measurement of spatial and/or temporal coupling between oculomotor control and UL motor control during an UL task.

**Results:**

Twelve articles were included with a total of 427 participants. Most of the included studies (75%) were high-quality papers, and the remaining ones were of moderate quality. Participant’s NDDs were mainly developmental coordination disorder, cerebral palsy or autism spectrum disorder. The UL tasks performed in these studies were categorized as pointing tasks, manipulating tasks or tracing and copying tasks. Eye-hand coordination temporal pattern did not differ between individuals with and without NDD in simple tasks involving direct pointing at a single stationary target. In the case of more complex tasks in terms of visuomotor and/or cognitive integration, especially for the more complex manipulation or sequential movements, individuals with NDD exhibited significantly different behaviors, with larger temporal gaps between the timing of eyes movement relative to that of the hand and more reliance on visual monitoring of hand movements.

**Conclusion:**

The results of this systematic review suggest that individuals with NDD face significant challenges in efficiently integrating visual and motor information during UL tasks that are visually, cognitively and/or physically more demanding, with more reliance on visual feedback control. These findings emphasize the importance of monitoring eye-hand coordination deficits in this population to further improve and tailor therapeutic interventions.

## Introduction

Visually-guided upper limb (UL) movements, such as intercepting, reaching, grasping, tracking or manipulating objects, requires the ability to visually analyze the environment and to precisely coordinates movement of both the eyes and the UL, which is referred to as eye-hand coordination (1–3). Eye-hand coordination depends on temporal and spatial coupling (4): eyes and hand movements are initiated by a common command signal (temporal), and both systems use a common representation of target location (spatial) (4, 5). This sensory-motor integration effectively supports visually guided tasks, such as catching a ball or threading a needle (6). Typically, the eyes start moving toward the target, arrive and depart earlier than the hand, reflecting an anticipatory behavior that uses visual cues to guide movement (7). This is especially evident in well-learned tasks like lifting familiar objects (8, 9). In contrast, in learning a novel motor task, the motor system relies more on real-time, online feedback (10). This highlights the adaptability of motor coordination characterized by ongoing equilibrium between feedforward and feedback control that depends on the level of precision and demands of motor task, as postulated by the Optimal Feedback Control theory (11, 12).

Eye-hand coordination during object manipulation has been shown to differ between children (under 10 years old) and adults (7, 13), with the coupling between oculomotor and manual systems evolving into a more efficient and mature pattern around the age of 10 years old (7). This is not surprising given the maturation of the numerous cerebral regions involved in eye-hand coordination during childhood, including the brainstem, basal ganglia, cerebellum, and the frontal and parietal cortices, as well as of the pathways between these regions (14, 15). As a result, lesions or developmental disorders affecting various cerebral regions or pathways can lead to impairments in eye-hand coordination and visually-guided UL movements (2, 16). Neurodevelopmental disorders (NDDs) are defined as a large group of deficits or delays that negatively affect the development of the child’s brain or nervous system, leading to significant changes in cognitive, social, and behavioral functioning that may cause impairments in their motor and sensory systems (17). NDDs include autism spectrum disorder (ASD), attention deficit hyperactivity disorder (ADHD), developmental coordination disorders (DCD), cerebral palsy (CP), intellectual disability (ID), communication disorders, specific learning disorder, and motor disorders (17). Individuals with NDDs often face difficulties in analyzing and responding to sensory information, including visual information, in order to produce coordinated movement, which may result in long-term challenges in daily-life functioning (18–22).

Despite the interdependence between the oculomotor and UL motor systems, eye movement behavior and UL movement behavior are often assessed separately, particularly for populations living with neurological conditions such as NDD (23). On the one hand, studies evaluating UL motor control in individuals with CP and DCD show significant disorders in motor planning and execution (24–26). Individuals with CP exhibit motor planning impairments, particularly in force scaling with the more affected hand, posture planning difficulties in object manipulation with both hands (24), and other execution issues like impairments in selective finger movements and difficulties to achieve various types of grasping, which impact on the UL function and affect daily activities and overall independence (25). Likewise, children with DCD face substantial challenges related to coordination, timing, and force modulation, particularly during tasks that necessitate synchronization between manual movements and external stimuli (26). These impairments in UL control serve as indicators of more extensive impairments in motor system, often influenced by atypical sensorimotor integration (27). On the other hand, there is evidence of oculomotor dysfunctions in NDD populations (28, 29). For example, children with CP show atypical saccadic (i.e., rapid ocular movement repositioning the fovea toward a location of interest) (23) and smooth pursuit eye movements (i.e., uninterrupted ocular movement provoked by an object in motion) (23, 29). Children with ASD frequently exhibit challenges in inhibiting saccades, visual smooth pursuit of a moving target and longer oculomotor fixation (i.e., maintaining stable gaze on a specific point in space while inhibiting ocular drifts) (30), which impact on their ability to process adequately visual information that guide their movements (28).

However, there is a growing number of studies focusing on the spatiotemporal aspects of eye-hand coordination across various NDDs (31–36). While individual studies suggests deficits compared to typically developing peers in various aspects of eye-hand coordination in populations of children living with an hemiplegic CP (31, 34), ASD (32) or DCD (33), there has been no attempt to compare eye-hand coordination patterns across NDDs to identify similarities or differences. This is particularly relevant given the considerable comorbidity and phenotypic overlap and the difficulty to establish etiology (37–39).

Understanding the challenges individuals with NDDs encounter when performing UL motor tasks that require accurate eye-hand coordination, such as predictive motor control, may help inform the development of targeted rehabilitation strategies. Recent research showed that training methods strengthening visuomotor integration and internal modelling can improve and enhance eye-hand coordination movement kinematics in children with DCD (40). Therefore, the present systematic review aims to compare eye-hand coordination patterns between individuals with or without a NDDs while performing UL motor tasks. More specifically, this study aims to characterize the differences in spatiotemporal eye-hand coordination in these populations in order to get a better insight into the motor strategies employed, i.e., whether gaze behavior in relation the hand movement suggests a more anticipatory behavior (gaze is directed toward desired end movement location such as a target or object) or a more feedback-dependent behavior (maintaining visual monitoring on the UL).

## Methods

The reporting of this systematic review was guided by the standards of the Preferred Reporting Items for Systematic Review and Meta-Analysis (PRISMA) Statement (41). The completed PRISMA 2020 checklist is provided in Appendix A. The protocol was registered on the PROSPERO platform under the number: CRD42024536941. The publicly accessible URL is: https://www.crd.york.ac.uk/PROSPERO/view/CRD42024536941.

### Selection criteria

The inclusion criteria for the studies were as follows:

1. Studies must include individuals diagnosed with a NDD. This includes both disorders formally classified under the Diagnostic and Statistical Manual of Mental Illnesses or DSM-5 (i.e., DCD, ASD, ADHD, intellectual disability, learning disorders), and other conditions of neurodevelopmental origin, such as CP or genetic disorders that are frequently associated with sensorimotor and cognitive impairments during development (42–44). If the sample includes individuals with diagnoses other than NDDs, the study has to present data in a way that allows the extraction of data for participants with NDDs. Given the fact that studies in this field are limited, both studies in children and adults were included. However, to limit the potential confounding effect of aging, only studies with young and middle-aged adults are included (<50 years old) (45);
2. Studies must include an aged-matched control group (CTRL) without NDD, and perform statistical comparisons of variables of interest between groups;
3. Studies must measure the spatial and/or temporal coupling between UL motor control and oculomotor control while performing an UL motor task;
4. Papers must be original, and peer-reviewed and employ a quantitative methodology with empirical data. Full text must be available in English or French.

The following study designs/publication types were excluded: qualitative studies, reviews, conference abstracts, case studies, or dissertations.

### Information sources and search

The development of the research strategy was elaborated by the research team in consultation with the librarian (MG). The following electronic databases were initially searched by the librarian (MG) for peer-reviewed articles in December 2022 and re-searched in April 2024: CINAHL Plus with Full Texts (EBSCO), EMBASE, WEB OF SCIENCE, MEDLINE (Ovid) and ERGONOMICS ABSTRACTS (EBSCO), using the following main concepts: oculomotor control, UL movement, eye-hand coordination, and NDDs. For each database, keywords were derived from these terms. The comprehensive search strategy employed for each database is delineated in Appendix B. There were no date limits in this review. No constraints were made on language, publication date, and participants’ age. The reference lists of the included papers were also scanned for additional relevant articles. The full search strategies of each database are presented in the Supplementary materials.

### Selection of sources of evidence and data extraction

Following PRISMA recommendations (41), the screening strategy was conducted in four steps:

1. Identification: the librarian (MG) performed the database search to identify the relevant records saved into EndNote software (Clarivate Analytics, Philadelphia, PA, USA). Then, the Covidence online software^1^ was used to remove duplicates and to perform the following two steps.
2. Screening: two independent reviewers (MA and IP) screened titles and abstracts based on the eligibility criteria.
3. Eligibility: two reviewers (MA and IP) independently reviewed the first 10% of full-text articles until reaching inter-rater agreement reflected by a reliability index (к ≥ 0 0.84) and then examined the full-text remaining articles. Reference lists of the selected papers were reviewed to find other eligible papers. Inclusion decision was made by consensus between the two reviewers. If needed a third reviewer (CM) was involved.
4. Data extraction: one reviewer (MA) extracted data, including the type of NDD, demographics of NDD and CTRL groups, experimental set-up, UL motor tasks description, eye-hand spatiotemporal coordination variables, and main findings comparing NDD group and CTRL group for eye-hand coordination variables.

### Critical appraisal of individual sources of evidence

Two reviewers (MA and IP) independently assessed the quality of the included studies using the 14-item quantitative research checklist developed by Kmet et al. (46), based on QualSyst manual guidelines. Each item was scored as “yes” = 2, “partial” = 1, “no” = 0, “n/a” = 1. A percentage score expressing article quality was calculated and classified as “Strong” (≥ 75%), “Moderate” (between 55 and 75%) or “Low” (≤ 55%) (46). An intraclass correlation coefficient (ICC) was calculated, yielding a value of 0.82, indicating a good level of agreement in the scoring of article quality by the reviewers. Disagreements was resolved by discussion, or by a third author (CM) if needed.

### Synthesis of results

The results of included studies were analysed according to a narrative-synthesis approach to show the similarities or differences in eye-hand spatiotemporal patterns of movement across different UL tasks between individuals with NDDs and CTRL individuals. The data was systematically classified based on the eye-hand coordination metrics used in the study:

1) Metrics of synchronization between eye and hand movements: the existence of significant differences between the NDD and CTRL groups was reported to assess the extent of synchronization.
2) Metrics of temporal delay between the timing of eyes movement relative to hand and the variability in their spatial trajectory: the existence of significant differences between the NDD and CTRL groups was reported in addition to the motor behavior adopted, that was classified as either:
  a) Feedforward behavior (anticipatory): the eyes movement lead the hand movement, indicating a reliance on anticipatory strategies.
  b) Feedback behavior (visual monitoring of UL preforming the motor action): the delay between eyes and hand movement is close to zero or the eyes lag behind the hand movement, indicating a dependence on feedback-driven visual monitoring.

## Results

**Figure 1:**
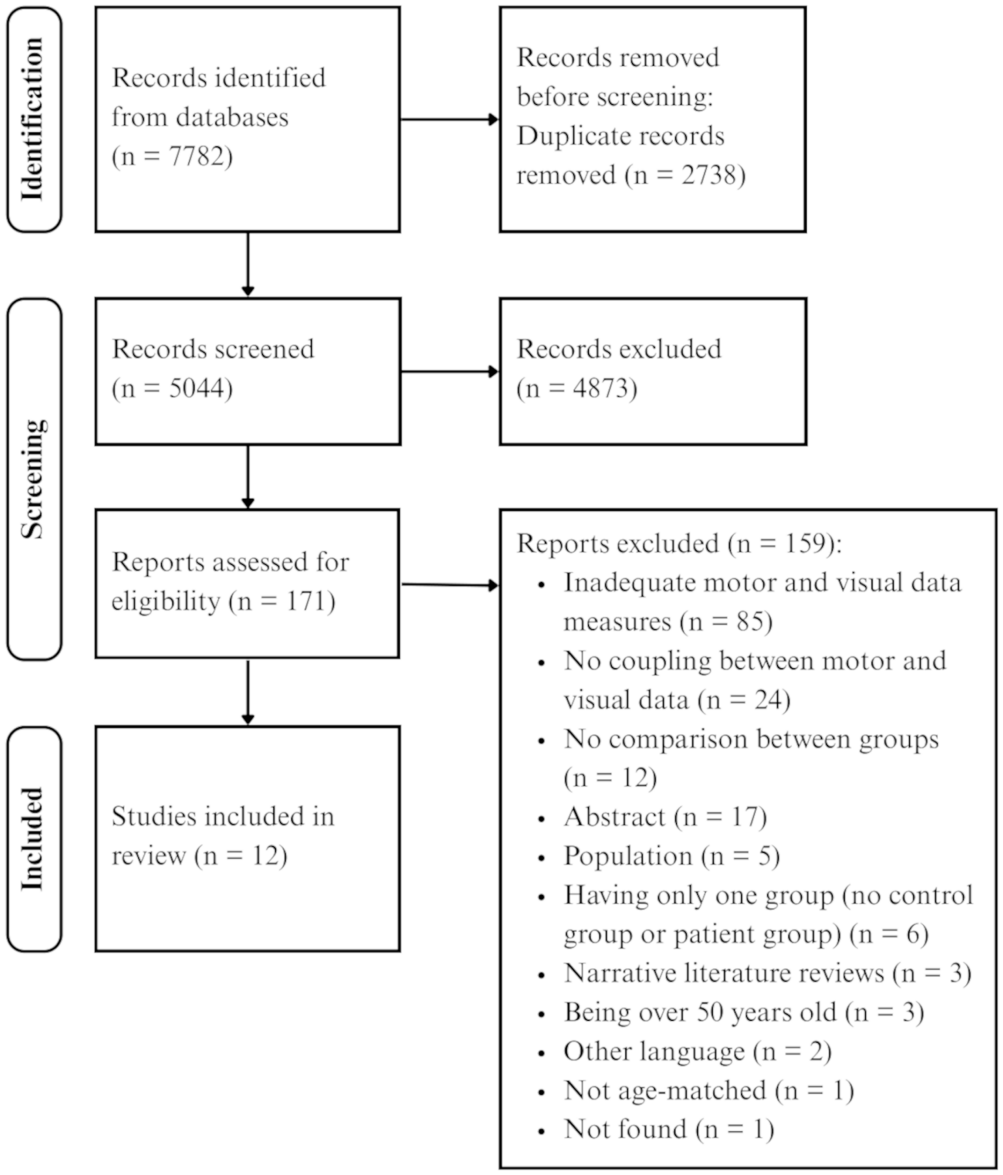
PRISMA flow diagram.

The search and study selection process are presented in Figure 1. The search through the five databases yielded 7,782 articles. After removing the duplicates, 5,044 records remained. After screening titles and abstracts, 4,873 were excluded. Of 171 articles that were analyzed for eligibility, 159 were excluded mainly because these studies did not record eye and hand movements but rather used standardized clinical tests, including tasks demanding eye-hand coordination. Therefore, a total of 12 articles published between 1994 and 2023 were included in this review.

### Study characteristics

The main characteristics of the included studies are outlined in Tables 1–3. When considering all included studies together, NDD groups include a total of 189 participants (mean age ranged from 3.1 to 40.8 years, with an average of 15.8 years; standard deviations were available for 9 studies, averaging 3.1 years, while 3 studies did not report this data; 110 males, 72 females, sex not reported for the remaining participants), and the CTRL groups include a total of 238 participants (mean age ranged from 3.1 and 37.05 years), with an average of 15.8 (based on 11 studies, one study noted age-matching without specifying means); the average standard deviation, reported in 9 studies, was 2.41 years; 114 males, 118 females (sex not reported for the remaining participants). NDD included developmental coordination disorder [DCD; 4 studies (33, 47–49)], cerebral palsy [CP; 3 studies (31, 34, 50)], autism spectrum disorder [ASD; 3 studies (35, 51, 52)], neurofibromatosis type I [NFI; 1 study (53)], and intellectual disability [ID; 1 study (54)]. The UL tasks used in the included studies can be classified according to the following categories: (1) pointing tasks, involving pointing on a screen using the hand or an object (such as a mouse); (2) object manipulation tasks (including natural playing with toys); and (3) tracing and copying tasks. These tasks varied from simpler to more complex ones based on the level of visual, cognitive and/or motor integration demands.

**Table 1:** Temporal and spatial eye-hand coordination variables and their definitions.

Variables (and measurement unit)	Definitions
Eye-hand movement onset asynchrony (MOA; in ms) (31, 33, 34, 47, 50, 51, 53)	Temporal gap between eye movement onset toward a target and the onset of a hand movement. Behavior is considered feedforward (anticipatory) when the eye movement onset leads that of the hand movement.
Eye-hand movement termination asynchrony (MTA; in ms) (33, 34, 49, 50)	Temporal gap between the end of a hand movement and the end of eye movement. Behavior is considered feedforward (anticipatory) when the eye movement termination leads that of the hand movement.
Foveation time (in ms) (33, 49, 51)	Temporal gap between the arrival of eye movement on a target and the hand movement onset time to the same target. Behavior is considered feedforward (anticipatory) when the arrival of eye movement on the target leads the hand movement onset to the same target.
Temporal buffering of information (in ms) (33)	Temporal gap between the end of hand movement to a first target and the onset of eye movement to a second target.
Eye-hand coupling (in ms) (49)	Temporal gap between the hand movement onset time on a target and the departure of eye movement to the same target. This value may reflect either eye departure occurring before hand initiation, or hand initiation occurring while gaze remains on the target.
Eye-hand movement correlation coefficient (35)	Covariance of the eye and hand movement coordinates divided by the product of their variances.
Vector correlation coefficient (54)	Covariation between gaze and penpoint movement (tracing and copying task).
Peak covariation (48)	Peak covariation between vertical hand and eye position from grasp onset until lift offset (object manipulation task).
Eye-hand time lag (in ms) (48)	Degree to which eye movements were preceding the hand at the time of Peak covariation (see previous variable). Behavior is considered feedforward (anticipatory) when the eye movement precede the hand movement.
Proportion of time in hand-eye Coordination (in %)_(52)	Proportion of time looking at an in-hand object compared to at non-manipulated objects.
Duration of looks during manipulation (in ms) (52)	Median duration of looks to in-hand object vs. non manipulated objects.
Looking behaviors before/after manual actions (52)	Likelihood of looking at the target object 3 s before/after a manual action.
Mean distance between eye mark and penpoint (in degrees) (54)	Distance was characterized as an angle (tracing and copying tasks).

**Table 2:** Characteristics of included studies using pointing tasks (*n* = 6).

Study	Sample	Set-up	Task and conditions	Variables (and measurement unit)	Main findings	Quality
Payne et al. (31)	CP: *N* = 8; 3F/5M; 12–39 yo; 23.6 ± 9.7 yo CTRL: *N* = 10; 3F/7M; NAv; 30.6 ± 4.2 yo	30° from the horizontal touchscreen, mouse and remote eye tracker	Pointing at 1 target with 5 conditions [manipulating initial eyes position, previous knowledge of target location and effector (hand/mouse)].	MOA in ms	The only significant difference between CP and CTRL groups across the 5 conditions was found when the eyes were starting from a different location than the hand: - Both groups displayed anticipatory oculomotor behavior with a longer delay for CP group in the condition in which a difference was found. CP: M_MOA_ = 106 ms (SE = 10 ms); CTRL: M_MOA_ = 68 ms (SE = 7 ms); 95% CI (Nav); *p* = 0.007	Strong
Bloch et al. (51)	ASD: *N* = 24; F10/14 M; 18–59 yo; 40.84 ± NAv yo CTRL: *N* = 24; F10/14 M; 19–58 yo; 37.05 ± NAv yo	Vertical screen, motion capture system and remote eye tracker	Pointing at 1 target to communicate it position (on the right or left) to the interaction partner in the presence of visual distractor with manipulation of initial eyes position	MOA in ms	Significant difference between ASD and CTRL groups: - Both groups displayed anticipatory oculomotor behavior, but with a longer delay for ASD group. ASD: M_MOA_ = 263 ms (SD = 103 ms); CTRL: M_MOA_ = 206 ms (SD = 60 ms); 95% CI (11.1; 110); *p* = 0.019	Strong
				Foveation time in ms	Significant difference between ASD and CTRL groups: - Both groups displayed anticipatory oculomotor behavior, but with a longer delay for ASD group. ASD: M_Foveation time_ = 127 ms (SD = 99 ms); CTRL: M_Foveation time_ = 75 ms (SD = 69 ms); 95% CI (Nav); *p* = 0.042	
Castricum et al. (53)	NFI: *N* = 22; F13/9 M; 18–55 yo; 28.9 ± 11 yo CTRL: *N* = 31; F19/12 M; 18–55 yo; 32.9 ± 11.1 yo	Vertical touchscreen and remote eye tracker	Pointing at 1 target with 4 conditions (at the opposite side a visual target, at its remembered position and intercepting a moving target by predicting it final position)	MOA in ms	No significant difference for any conditions: - Both groups displayed a similar anticipatory behavior. Pointing at direct target: NFI: M_EOM_ = 205 ms (SD = 22 ms); CTRL: M_EOM_ = 224 ms (SD = 20 ms); 95% CI (Nav); *p* < 0.05; NFI: M_HOM_ = 342 ms (SD = 42 ms); CTRL: M_HOM_ = 342 ms (SD = 40 ms); 95% CI (Nav); *p* (ns) Pointing at the opposite side: NFI: M_EOM_ = 269 ms (SD = 66 ms); CTRL: M_EOM_ = 319 ms (SD = 90 ms); 95% CI (Nav); *p* (ns); NFI: M_HOM_ = 426 ms (SD = 61 ms); CTRL: M_HOM_ = 415 ms (SD = 68 ms); 95% CI (Nav); *p* (ns) Pointing at its remembered position: NFI: M_EOM_ = 373 ms (SD = 193 ms); CTRL: M_EOM_ = 537 ms (SD = 222 ms); 95% CI (Nav); *p* < 0.05; NFI: M_HOM_ = 580 ms (SD = 75 ms); CTRL: M_HOM_ = 538 ms (SD = 73 ms); 95% CI (Nav); *p* (ns) Intercepting moving target: NFI: M_EOM_ = 471 ms (SD = 36 ms); CTRL: M_EOM_ = 495 ms (SD = 41 ms); 95% CI (Nav); *p* (ns); NFI: M_HOM_ = 800 ms (SD = 184 ms); CTRL: M_HOM_ = 754 ms (SD = 122 ms); 95% CI (Nav); *p* (ns)	Strong
Wilmut et al. (33)	DCD: *N* = 7; NAv; 7–8 yo; 7.5 ± NAv yo CTRL: *N* = 10; NAv; 7–8 yo; 7.8 ± NAv yo	Vertical touchscreen, remote eye tracker, motion capture system	Pointing at 2 sequential targets (visual/remembered second target)	MOA in ms	Trend for a difference between groups only for sequential pointing at the visual second target: - Both groups displayed anticipatory oculomotor behavior, but with a longer delay for DCD group. Visual second target: *Dominant hand:* DCD: M_MOA_ = 648 ms (SD = 225 ms); CTRL: M_MOA_ = 496 ms (SD = 97 ms); *Non-dominant hand:* DCD: M_MOA_ = 685 ms (SD = 187 ms); CTRL: M_MOA_ = 468 ms (SD = 84 ms); 95% CI (Nav); *p* = 0.055 for main effect of group; *p* (ns) for main effect of hand dominance	Moderate
				MTA in ms	Significant difference between groups for all conditions: - Both groups displayed anticipatory oculomotor behavior, but with a longer delay for DCD group. Visual second target: *Dominant hand:* DCD: M_MTA_ = 594 ms (SD = 43 ms); CTRL: M_MTA_ = 347 ms (SD = 57 ms); *Non-dominant hand:* DCD: M_MTA_ = 575 ms (SD = 110 ms); CTRL: M_MTA_ = 389 ms (SD = 111 ms); 95% CI (Nav); *p* < 0.01 for main effect of group; *p* (ns) for main effect of hand dominance Remembered second target: *Dominant hand:* DCD: M_MTA_ = 472 ms (SD = 62 ms); CTRL: M_MTA_ = 356 ms (SD = 86 ms); *Non-dominant hand:* DCD: M_MTA_ = 429 ms (SD = 79 ms); CTRL: M_MTA_ = 378 ms (SD = 137 ms); 95% CI (Nav); *p* < 0.05 for main effect of group; *p* (ns) for main effect of hand dominance	
				Foveation time in ms	Significant difference groups for all conditions: - Both groups displayed anticipatory oculomotor behavior, but with a longer duration for DCD group. Visual second target: *Dominant hand:* DCD: M_Foveation time_ = 275 ms (SD = 55 ms); CTRL: M_Foveation time_ = 74 ms (SD = 88 ms); *Non-dominant hand:* DCD: M_Foveation time_ = 257 ms (SD = 125 ms); CTRL: M_Foveation time_ = 157 ms (SD = 83 ms); 95% CI (Nav); *p* < 0.01 for main effect of group; *p* (ns) for main effect of hand dominance Remembered second target: *Dominant hand:* DCD: M_Foveation time_ = 225 ms (SD = 71 ms); CTRL: M_Foveation time_ = 167 ms (SD = 93 ms); *Non-dominant hand:* DCD: M_Foveation time_ = 210 ms (SD = 85 ms); CTRL: M_Foveation time_ = 129 ms (SD = 131 ms); 95% CI (Nav); *p* < 0.01 for main effect of group; *p* (ns) for main effect of hand dominance	
				Temporal buffering of information in ms	Significant difference between groups for all conditions: - Both groups displayed anticipatory oculomotor behavior, but with a longer duration for DCD group. Visual second target: *Dominant hand:* DCD: M_Temporal buffering_ = 221 ms (SD = 94 ms); CTRL: M_Temporal buffering_ = 104 ms (SD = 58 ms); *Non-dominant hand:* DCD: M_Temporal buffering_ = 180 ms (SD = 71 ms); CTRL: M_Temporal buffering_ = 149 ms (SD = 81 ms); 95% CI (Nav); *p* < 0.01 for main effect of group; *p* (ns) for main effect of hand dominance Remembered second target: *Dominant hand:* DCD: M_Temporal buffering_ = 152 ms (SD = 57 ms); CTRL: M_Temporal buffering_ = 148 ms (SD = 78 ms); *Non-dominant hand:* DCD: M_Temporal buffering_ = 155 ms (SD = 64 ms); CTRL: M_Temporal buffering_ = 98 ms (SD = 44 ms); 95% CI (Nav); *p* < 0.01 for main effect of group; *p* (ns) for main effect of hand dominance	
Wilmut et al. (47)	DCD: *N* = 23; 5F/18M; 6–23 yo; 12.9 ± NAv yo CTRL: *N* = 23; 13F/10M; 6–23 yo; 13 ± NAv yo	Horizontal touchscreen, remote eye tracker, motion capture system	Pointing at 1 target with conditions manipulating previous knowledge of target location: fixed cues (clear or ambiguous) and motion cues (with different cue duration, cue density, numbers of possible targets).	MOA in ms	Significant difference between groups for all conditions: Both groups displayed anticipatory oculomotor behavior, but with a longer delay for DCD group. Clear fixed peripheral cueing: DCD: M_MOA_ = 278 ms (SE = 66 ms); CTRL: M_MOA_ = 72 ms (SE = 25 ms); 95% CI (Nav); *p* < 0.001 Ambiguous fixed peripheral cueing: DCD: M_MOA_ = 356 ms (SE = 57 ms); CTRL: M_MOA_ = 163 ms (SE = 31 ms); 95% CI (Nav); *p* < 0.001 Clear fixed central cueing: DCD: M_MOA_ = 335 ms (SE = 63 ms); CTRL: M_MOA_ = 68 ms (SE = 38 ms); 95% CI (Nav); *p* < 0.001 Ambiguous fixed central cueing: DCD: M_MOA_ = 424 ms (SE = 67 ms); CTRL: M_MOA_ = 162 ms (SE = 32 ms); 95% CI (Nav); *p* < 0.001 Short-duration cueing (2 moving dots × 100 ms) with 4 possible targets: DCD: M_MOA_ = 372 ms (SE = 48 ms); CTRL: M_MOA_ = 194 ms (SE = 53 ms); 95% CI (Nav); *p* = 0.04 Longer-duration cueing (6 moving dots × 100 ms) with 4 possible targets: DCD: M_MOA_ = 292 ms (SE = 39 ms); CTRL: M_MOA_ = 39 ms (SE = 51 ms); 95% CI (Nav); *p* = 0.04 Short-duration cueing (2 moving dots × 100 ms) with 12 possible targets: DCD: M_MOA_ = 348 ms (SE = 59 ms); CTRL: M_MOA_ = 237 ms (SE = 82 ms); 95% CI (Nav); *p* < 0.001 Longer-duration cueing (6 moving dots × 100 ms) with 12 possible targets: DCD: M_MOA_ = 306 ms (SE = 50 ms); CTRL: M_MOA_ = 99 ms (SE = 34 ms); 95% CI (Nav); *p* < 0.001	Moderate
Zhang et al. (35)	ASD: *N* = 30; 9F/21M; 4–6 yo; 5.4 ± 0.7 yo CTRL: *N* = 30; 14F/16M; 4–6 yo; 5 ± 0.7 yo	Remote eye tracker and vertical screen	Pointing at multiple static and moving targets	Eye-hand movement correlation coefficient	Significant difference for both static and moving targets, with lower correlations in the ASD group. Static targets: ASD: M_Horizontal coordinate correlation_ = 0.84 (SD = 0.06); CTRL: M_Horizontal coordinate correlation_ = 0.96 ms (SD = 0.03); 95% CI (Nav); *p* < 0.001; ASD: M_Vertical correlation_ = 0.65 (SD = 0.14); CTRL: M_Vertical correlation_ = 0.84 (SD = 0.15); 95% CI (Nav); *p* < 0.001 Moving targets: ASD: M_Horizontal coordinate correlation_ = 0.54 (SD = 0.1); CTRL: M_Horizontal coordinate correlation_ = 0.77 ms (SD = 0.17); 95% CI (Nav); *p* < 0.001; ASD: M_Vertical correlation_ = 0.45 (SD = 0.15); CTRL: M_Vertical correlation_ = 0.65 (SD = 0.17); 95% CI (Nav); *p* < 0.001	Strong

NAv: non-available; N: sample size; F: female; M: male; age interval; mean ±SD; >/<: significant greater or lower value of eye-hand coordination outcomes for one group compared to other; MOA: eye-hand movement onset asynchrony; MTA: eye-hand movement termination asynchrony; CP: cerebral palsy; ASD: autism spectrum disorder; NFI: neurofibromatosis type I; DCD: developmental coordination disorder; CTRL: control group; M: mean; SD: standard deviation; SE: standard error; CI: confidence interval; EOM: eye onset movement; HOM: hand onset movement; ns: not significant.

**Table 3:** Characteristics of included studies using object manipulation (*n* = 5).

Study	Sample	Set-up	Task and conditions	Variables (and measurement unit)	Main findings	Quality
Arthur et al. (48)	DCD: *N* = 19; 4F/15M; 8–12 yo; 9.7 ± 1.2 yo CTRL: *N* = 39; 20F/19M; 8–12 yo; 9.6 ± 1.1 yo	Horizontal desk, motion-tracking camera and eye-tracking glasses	Grasping and lifting consecutively and randomly cylinders of identical height (7.5 cm) but varying diameters (5 cm, 7.5 cm, and 10 cm) and weights (355 g or 490 g)	Peak covariation between eye-hand movement	No significant difference between groups. DCD: Median_Peak Covariation_ = 0.38 (IQR = 0.06); CTRL: Median_Peak Covariation_ = 0.39 (IQR = 0.08); 95% CI (Nav); *p* = 0.47	Strong
				Eye-hand time Lag in ms	No significant difference between groups. Both groups display similar anticipatory oculomotor behavior. DCD: Median_Eye-hand lag_ = 80 ms (IQR = 178 ms); CTRL: Median_Eye-hand lag_ = 50 ms (IQR = 140 ms); 95% CI (Nav); *p* = 0.34	
Verrel et al. (50)	Hemiplegic CP: *N* = 6; 5F/1M; 14–19 yo; 16.1 ± 1.8 yo CTRL: *N* = 10; 9F/1M; 20–25 yo; NAv yo	Horizontal desk, motion-tracking camera and eye-tracking glasses	Grasping and transporting cylinders of matching colors from either side of a desk to the opposite side, with and without an obstacle in between	MOA normalized to hand movement duration (%)	CP group (more affected hand) significantly differs from CTRL group (either hand) and from CP group (less affected hand): - CP group (more affected hand): longer visual monitoring of the object being transported to its target [*no obstacle*: M_MOA_ = 13.4% (SD = 4.4%); *Obstacle*: M_MOA_ = 14.6% (SD = 2.1%)] - CP group (less affected hand): less visual anticipation [*no obstacle*: M_MOA_ = −6.9% (SD = 5.8%); *Obstacle*: M_MOA_ = 1.5% (SD = 5%)] - CTRL group (dominant hand): less visual anticipation [*no obstacle*: M_MOA_ = −4.5% (SD = 4.8%); *Obstacle*: M_MOA_ = 0.02% (SD = 4.2%)] - CTRL group (non-dominant): shorter visual monitoring [*no obstacle*: M_MOA_ = −3.1% (SD = 4.8%); *Obstacle*: M_MOA_ = 3.8% (SD = 4.4%)] Main effect hand: *p* < 0.002; Group-hand interaction: *p* < 0.005	Strong
				MTA normalized to hand movement duration (%)	CP group (more affected hand) significantly different from CTRL group (either hand) and from CP group (less affected hand): - CP group (more affected hand): less anticipation [*no obstacle*: M_MOA_ = −49.5% (SD = 2.8%); *Obstacle*: M_MOA_ = −51.4% (SD = 3.6%)] - CP group (less affected hand): more anticipation [*no obstacle*: M_MOA_ = −76.8% (SD = 5.4%); *Obstacle*: M_MOA_ = −61.2% (SD = 6.9%)] - CTRL group (dominant): more anticipation [*no obstacle*: M_MOA_ = −71.5% (SD = 4.6%); *Obstacle*: M_MOA_ = − % (SD = 4.2%)] - CTRL group (non-dominant hand): more anticipation [*no obstacle*: M_MOA_ = −75.7% (SD = 4.4%); *Obstacle*: M_MOA_ = −65.6% (SD = 3.8%)] Main effect hand: *p* < 0.0005; Group-hand interaction: *p* < 0.005	
Surkar et al. (34)	Hemiplegic CP: *N* = 13; 8F/5M; 3–7 yo; 4.9 ± 1.1 yo CTRL: *N* = 10; 9F/6M; 4–7 yo; 5.8 ± 1.1 yo	Horizontal board, head mounted eye tracker with scene and eye camera	Grasping and transporting objects to six target positions (from 0° to 180° clockwise and counterclockwise), based on visual cues, both ULs were evaluated, clockwise positions more complex for right UL and counterclockwise more complex for left UL.	MOA in ms	CP group significantly differs from CTRL group during the initial stages of the task (*reaching to grasp phase: from appearance of starting stimulus to starting movement toward the object and grasping phase: starting movement toward the object to start grasping the object*): - Both groups displayed anticipatory oculomotor behavior, but with a longer delay for CP group (with both the more affected and less affected hand). Reaching to grasp phase: CP: M_MOA_ = 765 ms (SD = 108 ms); CTRL: M_MOA_ = 250 ms (SD = 116 ms); 95% CI (Nav); *p* = 0.001 Grasping phase: CP: M_MOA_ = 480 ms (SD = 61 ms); CTRL: M_MOA_ = 154 ms (SD = 65 ms); 95% CI (Nav); *p* = 0.001 CP group significantly differs from CTRL group during the last stage of the task *(Transporting phase: from grasping to placing the object at a target position)* - CP group (both hands) displayed a feedback behavior (visual monitoring of the limb) while the CTRL group (both hands) displayed anticipatory oculomotor behavior. Transporting phase: CP: M_MOA_ = −220 ms (SD = 57 ms); CTRL: M_MOA_ = 121 ms (SD = 51 ms); 95% CI (Nav); *p* = 0.01	Strong
				MTA in ms	CP group (both hands) significantly differs from CTRL group (either hand): - CP group (both hands): longer visual monitoring of object being transported to it target before fixating the target - CTRL group (both hands): shorter visual monitoring of object being transported to it target before fixating the target CP: M_MTA_ = 356 ms (SD = 51 ms); CTRL: M_MTA_ = 189 ms (SD = 46 ms); 95% CI (Nav); *p* = 0.01	
Warlop et al. (49)	DCD: *N* = 6; 1F/5M; 20–23 yo; 21.8 ± 1.1 yo CTRL: *N* = 6; 1F/5M; 20–23 yo; 21.6 ± 1.2 yo	Horizontal desk, head-mounted eye-tracking and video recording of hand movement	Grasping and stacking 3 cups on a central target, alternating hands	Foveation time in ms	No significant difference between groups: - Both groups display similar anticipatory oculomotor behavior Grasping first cup: DCD: M_Foveation time_ = 285 ms (SD = 267 ms); CTRL: M_Foveation time_ = 251 ms (SD = 234 ms); 95% CI (Nav); *p* (ns) Stacking first cup: DCD: M_Foveation time_ = 68 ms (SD = 118 ms); CTRL: M_Foveation time_ = 89 ms (SD = 111 ms); 95% CI (Nav); *p* (ns) Grasping second cup: DCD: M_Foveation time_ = 186 ms (SD = 121 ms); CTRL: M_Foveation time_ = 172 ms (SD = 70 ms); 95% CI (Nav); *p* (ns) Stacking second cup: M_Foveation time_ = 0 ms (SD = 113 ms); CTRL: M_Foveation time_ = 79 ms (SD = 93 ms); 95% CI (Nav); *p* (ns) Grasping third cup: DCD: M_Foveation time_ = 118 ms (SD = 74 ms); CTRL: M_Foveation time_ = 65 ms (SD = 110 ms); 95% CI (Nav); *p* (ns) Stacking third cup: DCD: M_Foveation time_ = −14 ms (SD = 87 ms); CTRL: M_Foveation time_ = −23 ms (SD = 138 ms); 95% CI (Nav); *p* (ns)	Strong
				Eye-hand coupling in ms	Significant difference between DCD and CTRL groups: for stacking the second cup only: - Both groups displayed anticipatory oculomotor behavior, but with a longer delay for DCD group. Stacking second cup: DCD: M_Coupling_ = 492 ms (SD = 120 ms); CTRL: M_Coupling_ = 245 ms (SD = 105 ms); 95% CI (Nav); *p* = 0.006	
				MTA in ms	Significant difference between DCD and CTRL groups for grasping the third cup only: - Shorter visual monitoring for CTRL group - Anticipatory oculomotor behavior for DCD group Grasping second cup: DCD: M_MTA_ = −83 ms (SD = 33 ms); CTRL: M_MTA_ = −8 ms (SD = 49 ms); 95% CI (Nav); *p* = 0.028	
Yurkovic et al. (52)	ASD: *N* = 14; 6F/8M; 2–3 yo; 3.1 ± 0.5 yo CTRL: *N* = 15; 6F/8M; 2–3 yo; 3.1 ± 0.7 yo	Horizontal play area (carpet), head-mounted eye tracker and video recording	Naturalistic play with common toys using both hands (without specific instructions)	Proportion of Time in Hand-Eye Coordination in %	No significant difference between groups: - Both groups had a general preference for looking at non-manipulated toys over manipulated ones; 95% CI (Nav); *p* < 0.01 Manipulated toys: ASD: M_Time proportion_ = 40% (SD = 11%); CTRL: M_Time proportion_ = 41% (SD = 12%); 95% CI (Nav); 95% CI (Nav); *p* = 0.92 Non-manipulated toys: ASD: M_Time proportion_ = 52% (SD = 11%); CTRL: M_Time proportion_ = 51% (SD = 8%); 95% CI (Nav); 95% CI (Nav), *p* = 0.77	Strong
				Duration of Looks During Touch in ms	No significant difference between groups: - Both groups had longer time looking at manipulated toys than non-manipulated ones; 95% CI (Nav); *p* < 0.01 Manipulated toys: ASD: M_Looking duration_ = 2070 ms (SD = 1,460 ms); CTRL: M_Looking duration_ = 1760 ms (SD = 810 ms); 95% CI (Nav); 95% CI (Nav); *p* = 0.49 Non-manipulated toys: ASD: M_Looking duration_ = 720 ms (SD = 110 ms); CTRL: M_Looking duration_ = 760 ms (SD = 310 ms); 95% CI (Nav); 95% CI (Nav), *p* = 0.67	
				Looking Behaviors at the Moments Before/After Manual Actions	No significant difference between groups in the probability of looking at manipulated toys before/after action: - Both groups display similar anticipatory oculomotor behavior. Generalized linear mixed-effects model: F (1,27) = 2.91; corrected *p* = 1	

NAv: non-available; N: sample size; F: female; M: male; age interval; mean ±SD; >/<: significant greater or lower value of eye-hand coordination outcomes for one group compared to other; MOA: eye-hand movement onset asynchrony; MTA: eye-hand movement termination asynchrony; CP: cerebral palsy; DCD: developmental coordination disorder; ASD: autism spectrum disorder; CTRL: group control; M: mean; SD: standard deviation; CI: confidence interval; IQR: interquartile range; ns: not significant.

Table 1 presents the temporal and spatial eye-hand coordination variables assessed in the included studies, as well as their definitions. Most included studies focused on temporal aspects of eye-hand coordination (*n* = 11, vs. 1 study including 1 spatial variable). The most frequently reported variables are eye-hand movement onset asynchrony (MOA) (7 studies) (31, 33, 47, 50, 51, 53) and movement termination asynchrony (MTA) (33, 34, 49, 50). Please note that the methods used to measure those variables slightly varied across studies, with some studies looking at the initiation of saccade versus at the initiation of fixation.

The quality score of most studies was strong, with only three studies classified as moderate (33, 47, 54) (see Tables 2–4 and Appendix C). The two next sections present the results on the effect of NDD on spatiotemporal aspects of eye-hand coordination for each of the specific UL task categories.

**Table 4:** Characteristics of included study using tracing and copying task.

Study	Sample	Set-up	Task and conditions	Variables (and measurement unit)	Main findings	Quality
Kamoun et al. (54)	ID: *N* = 17; 8F/9 M; NAv; 15.5 ± 0.8 yo CA: *N* = 15; 7F/8M; NAv; 8.9 ± 1 yo MA: *N* = 15; 7F/8M; NAv; 15.2 ± 0.8 yo	Eye-mark recorder goggle unit, VHS videotape	- Tracing a visible line with a pen (i.e., with visual feedback) and then tracing the same line with no visual feedback as quickly as possible - Copying a drawing from a model and then reproducing it from memory	Mean distance between eye mark and penpoint in degrees	Significant differences between groups for all tasks: - Tracing with visual feedback [ID<MA (95% CI (Nav), *p* < 0.001) and CA (95% CI (Nav), *p* < 0.05)] ID: M_Mean distance_ = 2.6° [SD (Nav)]; MA: M_Mean distance_ = 3.8° [SD (Nav)]; CA: M_Mean distance_ = 3.4° [SD (Nav)] - Tracing without visual feedback [ID<MA (95% CI (Nav), *p* < 0.01)] ID: M_Mean distance_ = 2.7° [SD (Nav)]; MA: M_Mean distance_ = 3.6° [SD (Nav)] - Memory task [ID<CA (95% CI (Nav), *p* < 0.01)] ID: M_Mean distance_ = 4.1° [SD (Nav)]; CA: M_Mean distance_ = 6.1° [SD (Nav)]	Moderate
				Vector correlation coefficient	Significant difference between groups only for the invisible tracing task [ID<CA (95% CI (Nav), *p* < 0.05); M and SD (Nav)]	

NAv: non available; N: sample size; F: female; M: male; age interval; mean ±SD; > / <: significant greater or lower value of eye-hand coordination outcomes for one group compared to other; intellectual disability (ID); CA: chronological age group; MA: Mental age group; M: mean; SD: standard deviation; CI: confidence interval.

#### Effect of NDD on spatiotemporal aspects of eye-hand coordination

##### Pointing tasks

Table 2 shows the results of six studies that used pointing tasks with varying levels of complexity (31, 33, 35, 47, 51, 53). All these studies examined the temporal coordination between eyes and hands, and most of them assessed MOA.

Three studies [one high-quality (31) and two moderate-quality (33, 47)] investigated MOA in simple and direct pointing toward a single target. Despite the differences in terms of diagnosis, age and sample size between studies, the results show consistent results, i.e., no significant difference between the CTRL group and the NDD groups (either DCD or CP) (31, 33, 47). In all cases, the eyes consistently led the hand in reaction time toward the target for both groups (MOA), reflecting an anticipatory gaze behavior (31, 33, 47). Two of the studies instructed participants in both groups (DCD/CP and CTRL) to use only their preferred hand to perform the task (31, 33). Only one study compared the use of both hands in both groups (DCD and CTRL) and found no significant effect of hand dominance (33). In addition to MOA, this same study assessed foveation time and MTA (33). The findings showed that the eyes landed on the target before the hand onset time in both groups, indicating a consistent anticipatory pattern across participants (33). However, individuals with DCD exhibited significantly longer foveation times compared to the CTRL group (33). Similarly, for MTA, the hand landed on the target after the eyes, with significantly delays observed in children with DCD (33). In addition to their standard pointing tasks, two of these studies looked at whether modulating the predictability of target location influenced eye-hand coordination (31, 33). When the visual cue became more ambiguous, and the number of possible target positions increased, individual with DCD exhibited significantly longer MOA than the CTRL group, i.e., more anticipatory oculomotor behavior (33). No such effect was observed in another study using a somewhat similar task in CP (31). However, in this last study the sample size was smaller, and the CP group exhibited much diversity in terms of age and brain lesion localization (31).

In more complex tasks in terms of motor planification and execution, such as sequential movement toward two targets [studied in one moderate quality study (33)], children and young adults with DCD had a significantly longer MOA and MTA as well as longer foveation periods, i.e., all these metrics reflecting a more anticipatory oculomotor behavior in relation to the hand in individuals with DCD. Interestingly, this increase in MOA and MTA was more pronounced for the second target compared to the first in the sequential movement condition (33). This study also reported an increase in buffering time for the DCD group, a metric that represents the temporal gap between the completion of the hand movement at the first target and the initiation of the eye movement toward the second target (33). Another high-quality study conducted among children with ASD involved two complex pointing tasks, one toward multiple stationary targets and another toward moving targets (35). Results showed that individuals with ASD had significantly lower correlation coefficients between eye and hand movements for these two complex tasks compared to the CTRL group (35).

Overall, despite the heterogeneity in population characteristics across studies on pointing tasks, the eye-hand coordination metrics reveal similar anticipatory behavior across NDD and CTRL groups for very simple aiming and pointing tasks. However, for more complex tasks involving movement sequences, multiple targets, etc., differences were found between NDD and CTRL groups. The eye-hand coordination patterns were still anticipatory in both groups, but the NND group generally showed longer delays (for either MOA, MTA and foveation) or a decreased correlation between eyes and hand movements.

##### Object manipulation tasks

Table 3 presents the results of five high-quality studies that used object manipulation tasks ranging from simple object lifting to naturalistic play scenarios (34, 48–50, 52). All studies evaluated the temporal coordination between the eyes and hand, and the predominant metrics calculated were MOA and MTA. Some studies analyzed entire movement sequences while others focused on specific phases of the movement, offering in-depth analysis of specific aspects of object manipulation.

One study investigated grasping and lifting the same object but with different sizes and analyzed only the lifting phase (48). The results showed that eye-hand movements are moderately synchronized similarly for both groups, as measured by peak covariation with mean values close to 0.4, and that there is no significant difference between groups. The authors also measured the eye-hand time lag, which evaluates the general temporal delay between eye-hand movements throughout the entire analysed phase of movement. The results show that the eyes lead the hand movement during the entire lifting phase, i.e., anticipatory gaze behavior, but revealed no difference between groups. Note that this variable does not capture the beginning of the movement (it only considers the lifting phase), which might explain the apparent discrepancy with other results presented below for the MOA.

Three studies performed not only object grasping and lifting, but also target-directed placing of the object with increasing levels of complexity (34, 49, 50).

Two studies investigated eye-hand coordination in children with CP using grasping and transport tasks (34, 50). Surkar et al. (34) studied all the phases of movement of a sequential task involving reaching, grasping and transporting objects to six target positions, while Verrel et al. (50) analyzed only the transport phase to one of two target positions. Both studies reported group differences in MOA and MTA (50). Verrel et al. (50) found an effect of dominance and therefore reported results separately for each hand, while Surkar et al. (34) and found no effect of dominance and therefore pooled data from both hands. Surkar et al. found that hemiplegic CP and CTRL groups both showed anticipatory gaze behavior during the initial reaching phase as well as during the grasping phase of the task, but with longer delays between eyes and hand movement in the CP group (34). Both studies assessed MOA during the object transport phase, defined as the time between the initiation of hand movement to transport the object and the gaze leaving the object (34, 50). Both studies showed that during this phase, CTRL children shifted their gaze to the target before initiating hand movement toward it, demonstrating anticipatory gaze behavior, while children with CP generally began moving their hand toward the target before the gaze shifted away from the object, indicating prolonged visual monitoring (feedback strategy) (34, 50). Both studies also evaluated temporal eye-hand coordination at the end of the transport phase, i.e., when the object is placed on the target (MTA) (34, 50). These two studies used different task paradigms: one paradigm solicited more feedforward control, while the other study’s task required more feedback control (34, 50). However, in both cases, there was a significant difference between the CP and CTRL groups. The CP group tended toward a feedback strategy rather than a feedforward one [in one study, the CP group was less anticipative (50), and in the other, they adopted a more visual control strategy (34)].

The third study (49) investigated eye-hand coordination in individuals with and without DCD in a task involving grasping, transporting, and stacking three cups on a central target. They measured foveation time, eye-hand coupling, and MTA across five sequences alternating between grasping and placing cups. Foveation time (the interval between eye fixation on a cup/target and hand movement initiation toward that same cup/target) showed no difference between groups but revealed considerable variability. For the other two variables (eye-hand coupling and MTA), differences were found only in one movement phase out of five, suggesting minor differences in eye-hand coordination. When differences were found, they reflected more anticipatory behavior for DCD group when placing the cup #2 on the target and shorter visual monitoring for CTRL group versus anticipatory behavior for DCD group when grasping cup #3.

Only one study used a more naturalistic task (free play with toys) in children with ASD (52). The results showed within-group effects of the condition (e.g., general preference for looking at other non-manipulated toys more than in-hand toy and gaze preceded manual action, indicating a similar anticipatory strategy between groups), but no significant difference between groups or interaction between group and condition.

In summary, the eye-hand coordination did not differ between NDD and CTRL groups when it comes to simple object lifting. However, anticipatory deficits were found between in individuals with NDD when investigating more complex interactions with objects, particularly regarding the timing of the end of eye movement relative to hand movement in the NDD group. Strategies in NDD individuals were characterized by more anticipatory behavior when starting object grasping phase and more reliance on visual monitoring of hand when starting to transport the object, and a tendency toward feedback than feedforward control was found regarding the end of timing of eyes relative to hand movement when finishing an object manipulation task.

##### Tracing and drawing tasks

Only one study used tracing and copying tasks, and it was the only included study investigating individuals with an ID as presented in Table 4 (54). It was also the only study that included a spatial assessment of eye-hand coordination. The results showed a larger distance between the gaze and hand paths across all tasks and conditions except model copying task in the group with an ID compared to two CTRL groups (one in which the chronological age was matched, and another in which the mental age was matched).

## Discussion

The present systematic review is the first to investigate eye-hand coordination between individuals with and without NDD. Twelve articles were included, 75% of which being high quality papers. The results reveal that eye-hand coordination deficits are task-dependent: eye-hand coordination was altered in individuals with NDD in UL motor tasks that were complex in terms of visual, cognitive and/or motor processing, while it was generally preserved for very simple tasks. Indeed, all the studies focusing on reaching to point a simple target found no significant difference between groups (31, 33, 47), while differences were observed when reaching to grasp an object (34, 49, 50). Studies evaluating tasks involving grasping and transporting objects also reported differences between groups, characterized by more anticipatory gaze behavior when initiating the object grasping phase and by more reliance on visual monitoring of hand when initiating the object transport phase (34, 50). Only one study investigated eye-hand coordination in a tracing and copying task and revealed differences between groups, with an increased dependence on visual monitoring (54).

Notable differences in the results were observed according to whether the aim of the reaching movement was to point a target or to grasp an object. The fact that individuals with NND did not differ from controls (both exhibiting a largely anticipatory gaze behavior) when reaching to point a stationary target (31, 33, 47) might be explained by the fact that such a task does not demand complex manipulation or force application and requires less spatial adjustments and use of ongoing feedback (55, 56). Unlike pointing, reaching to grasp requires higher precision and adaptability, implicating early trajectory corrections and continuous reliance on visual feedback (56, 57). Our results show that individuals with NDD display more anticipatory gaze behavior than the CTRL group, as reflected by longer delays between eyes and hand movements (34, 49, 50). Similar differences between groups have also been reported during the object grasping and transporting phases, particularly in individuals with CP (34, 50). They exhibit an extended temporal delay between the movement of the eyes and that of the UL compared to their typically-developing peers (34, 50). During reaching and grasping phases, a more anticipatory gaze behavior might be used to allow additional time to process the object features (e.g., shape, orientation, size and texture), to plan the movement and to secure the grip before lifting the object in an effort to overcome motor planning and somatosensory deficits (24, 58–62). During the transporting phase, the presence of somatosensory deficits is likely to explain why individuals with CP shows a shift in their strategy, transitioning from a more anticipatory gaze behavior to a close visual monitoring of their moving UL. For instance, individuals with CP tend do display excessive grip force, increasing their safety margin, and to have more difficulties in scaling their forces in advance to object weight or texture (63). These deficits appears to be more important when somatosensory function is poorer, and are believed to results, at least partially, from sensorimotor integration impairments (63).

Other studies have investigated some specific task conditions that introduce greater complexity by increasing the demands on visuomotor and cognitive integration in individuals with NDDs, including CP, ASD, DCD and ID (31, 33, 35, 47, 51, 54). Tasks that involve targeting sequential (33) or multiple points (35), manipulating initial eyes position (31), previous knowledge of target location (31, 47) and copying tasks based on memory (54), add significant layers of cognitive load, which makes eye-hand coordination more challenging. Results when performing this kind of tasks often showed more anticipatory gaze behavior (presumably reflecting a longer motor planning process) (31, 33), reduced synchronization between eyes and hand movements (35), or an over-reliance on visual monitoring in individuals with NDD compared to controls (54). To explain task-dependent differences in gaze behavior, researchers developed a computational framework categorizing eye-hand coordination into two modes according to the task’s complexity: the common mode and the separate mode (64). The common mode is suitable for simpler tasks, in which the eyes and hand movements are closely coupled to follow a unified motor plan (64). The separate mode is essential for handling more complex tasks, requiring independent coordination between the eyes and hand (64). This mode is adopted, for example, in the case of a sequential pointing tasks where the eyes move and fixate the next target before the hand completes the current one (64, 65). This indicates that the effectors work independently rather than synchronously (64, 65). Results of this review suggest that this mode is particularly challenging for persons with NDDs due to their difficulties in managing the increased cognitive load and the independent control of each effector. An interesting parallel can be made with bimanual coordination, which can also involve a closely coupled control (while performing bimanual symmetric tasks, such as carrying a tray) or an independent control (while performing bimanual asymmetric tasks, such as cutting a steak with a knife and fork) of two effectors. It has been shown that individuals with CP exhibit more deficits in asymmetrical bimanual tasks compared to symmetrical ones (66).

The results of this review demonstrated that hand dominance has a different impact on eye-hand coordination depending on the nature of NDDs (33, 50). In DCD, similar alterations in eye-hand coordination are observed no matter the hand tested (33). However, this is not the case in population with an asymmetry in motor function between both ULs as in the case of hemiparetic CP (50). This asymmetry requires managing two very distinct internal motor models of each UL (67–69). When there is a need to apply the common mode of control between eyes and both hands in bimanual task, the nervous system may encounter difficulties in applying a shared control strategy due to this asymmetry (64). Given the challenge of performing ‘two tasks at once’, some authors have proposed the use of visually-coupled feedback to facilitate bimanual tasks in individuals with CP (70). The present review highlighted a research gap in the study of coordination between the oculomotor and UL systems in bimanual tasks, with only one included paper attempting to explore this topic (49). However, this study was incapable of providing conclusive results because of the variability in spatiotemporal eye-hand coupling translating the complexities in assessing bimanual coordination. Further research is needed to address this topic given the fact that most everyday activities require the visually-guided coordinated use of both ULs.

## Limitations

The findings derived from this present systematic review should be interpreted with consideration of some limitations. Because of the heterogeneity in participant profiles (age range and various types of NDDs), task demands and levels of complexity, as well as the use of different operational definitions to measure the same variable across studies, the conduct of a meta-analysis was not feasible. A narrative synthesis was preferred to allow for group comparisons while preserving contextual nuances. There is a bias of language in this review, that could exclude some relevant studies that were published in other languages other than French or English. In addition, the generalizability of our results is limited due the heterogeneity in age range, task demands and sample sizes. Furthermore, drawing conclusions for this review was challenging due to the inclusion of various NDDs. Another limitation of the studies included in this review is that most of them (10 articles) did not assess oculomotor control independently, in tasks not involving UL (e.g., simple saccade or smooth pursuit tasks), which makes it difficult to disentangle the potential contribution of oculomotor deficits to the observed deficits in eye-hand coordination. The two studies that assessed oculomotor control showed that the oculomotor performances of individuals with NDD were similar to those of the control group (49, 53). Furthermore, the fact that eye-hand coordination was not reported as altered in simple pointing tasks suggest that the deficits observed in complex tasks do not simply reflects deficits in the control of saccades. However, a notable limitation in the reviewed studies is that these tasks were often performed in distinct studies. Directly comparing the effect of simpler and more complex tasks on eye-hand coordination would provide a better understanding of the contribution of motor vs. cognitive factors.

### Recommendations for rehabilitation practice and future research

The results of this review point to a need for adapting current rehabilitation strategies for individuals with NDDs, particularly to better address the complexity of real-life tasks. It is recommended that rehabilitation protocols incorporate exercises that progressively increase task demands, such as cognitive challenges, time constraints, sequential movements, challenges to inhibitory control and working memory, to improve sensorimotor control and functional autonomy in individuals with NDD. More future research is warranted to understand underlying neurophysiological and cognitive mechanisms contributing to eye-hand coordination deficits in this population. It would be also important to conduct longitudinal studies that track the change in visuomotor control.

## Conclusion

This systematic review highlighted the challenges faced by population living with NDD to coordinate their oculomotor and UL motor systems in space and time, especially in more complex tasks. In simpler direct aiming, the findings demonstrated that individuals with NDD showed similar patterns of eye-hand movements to CTRL group. However, in more complex tasks, and particularly for manipulation tasks, they showed either more anticipatory gaze behavior (suggesting that they need more time to process the visual information required for the up-coming movement) or other adopted a feedback-driven behavior characterized by the ongoing visual monitoring of the moving UL (suggesting the use of compensatory strategies for somatosensory deficits).

## Data Availability

The original contributions presented in the study are included in the article/Supplementary material, further inquiries can be directed to the corresponding author.

## Footnotes

^1^ https://www.covidence.org/
