# Supplementary material for: Eye-hand coordination during upper limb motor tasks in individuals with or without a neurodevelopmental disorder: a systematic review
Source: Front Neurol. 2025 Jun 25;16:1569438. doi: 10.3389/fneur.2025.1569438 (PMC12238595; doi:10.3389/fneur.2025.1569438)
Supplement: Supplementary file 2 [file Data_Sheet_2.docx]

**Appendix B : Assessment of study quality using the quantitative research checklist of Kmet et al (2004)**

| **Study/Criteria** | **Q1** | **Q2** | **Q3** | **Q4** | **Q5** | **Q6** | **Q7** | **Q8** | **Q9** | **Q10** | **Q11** | **Q12** | **Q13** | **Q14** | **Score (%)** | **Quality** |
| --- | --- | --- | --- | --- | --- | --- | --- | --- | --- | --- | --- | --- | --- | --- | --- | --- |
| Payne et al (2017) | 2 | 1 | 0 | 2 | N/A | N/A | N/A | 2 | 1 | 2 | 2 | 1 | 2 | 2 | 17/22 (77.3%) | Strong |
| Bloch et al (2023) | 2 | 2 | 2 | 2 | N/A | N/A | N/A | 2 | 2 | 2 | 2 | 2 | 2 | 2 | 22/22 (100%) | Strong |
| Castricum et al (2023) | 2 | 1 | 2 | 1 | N/A | N/A | N/A | 2 | 2 | 2 | 2 | 2 | 2 | 2 | 20/22 (90.9%) | Strong |
| Wilmut et al (2006) | 1 | 1 | 2 | 2 | N/A | N/A | N/A | 1 | 1 | 1 | 2 | 2 | 1 | 2 | 16/22 (72.7%) | Moderate |
| Wilmut et al (2008) | 1 | 1 | 1 | 2 | N/A | N/A | N/A | 1 | 1 | 1 | 2 | 2 | 2 | 2 | 16/22 (72.7%) | Moderate |
| Zhang et al (2021) | 2 | 2 | 2 | 2 | N/A | N/A | N/A | 2 | 2 | 1 | 2 | 2 | 2 | 2 | 21/22 (95.5%) | Strong |
| Arthur et al (2021) | 2 | 1 | 2 | 2 | N/A | N/A | N/A | 2 | 1 | 2 | 2 | 2 | 2 | 2 | 20/22 (90.9%) | Strong |
| Verrel et al (2008) | 2 | 1 | 2 | 1 | N/A | N/A | N/A | 2 | 1 | 1 | 2 | 2 | 2 | 2 | 18/22 (81.8%) | Strong |
| Surkar et al (2018) | 2 | 1 | 2 | 2 | N/A | N/A | N/A | 2 | 1 | 1 | 2 | 1 | 2 | 2 | 18/22 (81.8%) | Strong |
| Warlop et al (2020) | 2 | 1 | 2 | 2 | N/A | N/A | N/A | 1 | 1 | 1 | 2 | 1 | 2 | 2 | 17/22 (77.3%) | Strong |
| Yurkovic et al (2021) | 2 | 1 | 1 | 2 | N/A | N/A | N/A | 2 | 1 | 1 | 2 | 1 | 2 | 2 | 17/22 (77.3%) | Strong |
| Kamoun et al (1994) | 1 | 1 | 1 | 2 | N/A | N/A | N/A | 2 | 1 | 1 | 1 | 1 | 2 | 2 | 15/22 (68.2%) | Moderate |

Legend: Design requirement: Q1: Question/objective sufficiently described?; Q2: Study design evident and appropriate?; Q3: Method of subject/comparison group selection or source of information/input variables described and appropriate?; Q4: Subject (and comparison group, if applicable) characteristics sufficiently described?; Q8: Outcome and (if applicable) exposure measure(s) well defined and robust to measurement / misclassification bias? Means of assessment reported?; Q9: Sample size appropriate?; Q10: Analytic methods described/justified and appropriate?; Q11: Some estimate of variance is reported for the main results?; Q12: Controlled for confounding?; Q13: Results reported in sufficient detail?; Q14: Conclusions supported by the results?
